# Bilingualism and Healthy Aging: Onset Age of Bilingualism as a Predictor of Older Adult Theory of Mind

**DOI:** 10.1093/geroni/igab046.1847

**Published:** 2021-12-17

**Authors:** W Quin Yow, Xiaoqian Li, Jia Wen Lee

**Affiliations:** 1 Singapore University of Technology & Design, Singapore, Singapore; 2 Singapore University of Technology and Design, Singapore, Singapore; 3 Singapore University of Technology & Design, Singapore University of Technology & Design, Singapore

## Abstract

The ability to understand and speak more than one language (i.e., bilingualism) may protect against age-related cognitive deterioration (Abutalebi et al., 2015). While there is mounting evidence suggesting that bilingualism confers advantages in domain-general cognitive abilities in late adulthood (see Bialystok, 2017, for a review), few studies have investigated the influences of bilingualism on socio-cognitive abilities such as theory of mind (ToM) in the normal aging process. Thus, in this study, we examine how bilingualism factors (i.e., onset age of bilingualism, language balance, and diversity in language use) are associated with individual differences in ToM in healthy older adult bilinguals aged 58-79 (N=44). ToM abilities were assessed using the Theory-of-Mind Task Battery (Hutchins et al., 2008), where participants viewed vignettes and answered questions about the protagonists’ cognitive and affective mental states. All participants completed a self-report language background questionnaire and the Montreal Cognitive Assessment (MoCA) test as a measure of general cognitive ability. Results revealed that better ToM was negatively correlated with participants’ chronological age (r=-.43, p=.004) and the onset age of second-language acquisition (r=-.41, p=.006), but not language balance and diversity (ps>.40). Partial regression analyses showed that earlier onset age of bilingualism predicted better ToM performance (β=-.40, p=.009), even after controlling for age, education, and general cognitive ability. These findings suggest that bilingual language experience, particularly earlier exposure to a second language, may provide benefits to older adults in preserving their ability to understand others’ mental states, acting as a cognitive reserve against age-related declines in socio-cognitive functions.

